# Food and Its Adulterations

**Published:** 1855-10

**Authors:** Arthur Hill Hassall


### SELECTIONS.

The following article from the distinguished author of the "Microscopic Anatomy of the Human Body, in Health and Disease," will be found exceedingly interesting and profitable. How far the facts here exhibited may apply to the American trade, we are not prepared to say; but if we are allowed to judge, from the notorious adulteration of medicinal agents, it is not unfair to conclude, that the revelations made by Dr. Hassall's Microscope across the water, would not be found very different from the state of things existing with us, could we have the focus of his Microscope nicely adjusted to the eye of the American public. What we here publish, is an abstract from the Virginia Medical & Surgical Journal, of the original articles which first appeared in the Quarterly Review for April, 1855.—*Editors Atlanta Medical & Surgical Journal.*

---

*Food and its Adulterations.* By Arthur Hill Hassall, M. D.

A story is told of an European who, wishing to convince a Brahmin of the folly of his faith in interdicting, as an article of food, anything that once poosessed life, showed him by the aid of the microscope, that the very water which he drank was full of living things. The Indian, thus suddenly introduced to an unseen world, dashed the instrument to the ground, and reproached his teacher for having so wantonly destroyed the guiding principles of his life. We too have at home a Hindoo, in the shape of the believing British public, to whose eye Dr. Hassall nicely adjusts the focus of his microscope, and bids him behold what unseen villanies are daily perpetrated upon his purse and person.

The world at large has almost forgotten Accum's celebrated work, "Death in the Pot;" a new generation has indeed sprung up since it was written, and fraudulent tradesmen and manufactures have gone on in silence, and, up to this time, in security, and falsifying their food and picking the pockets of the people. Startling indeed as were the revelations in that remarkable book, yet it had little effect in reforming the abuses it exposed. General denunciations of grocers did not touch individuals of the craft, and they were consequently not driven to improve the quality of their wares. The "Lancet" Commission went to work in a different manner. In Turkey, when of old they caught a baker giving false weight or adulterating the staff of life, they nailed his ear to the doorpost, "poua encourager les autres." Dr. Hassall, like a modern Al Rachid, perambulated the town himself, or sent his trustworthy agents to purchase articles, upon all of which the inexorable microscope was set to work, and every fraudulent sample, after due notice given, subjected its vendor to be pinned for ever to the terrible pages of the commissioner's report. In this manner direct responsibility was obtained. If the falsification denounced was not the work of the retailer, he was glad enough to shift the blame upon the manufacturer, and thus the truth came out.

A gun suddenly fired into a rookery could not cause a greater commotion than this publication of the names of dishonest tradesmen; nor does the daylight, when you lift a stone, startle ugly and loathsome things more quickly than the pencil of light, streaming through a quarter-inch lens, surprise in their naked ugliness the thousand and one illegal substances which enter more or less into every description of food that it will pay to adulterate. Nay, to such a pitch of refinement has the art of falsification of alimentary substances reached, that the very articles used to adulterate are adulterated. And while one tradesman is picking the pockets of his customers, a still more cunning rogue is, unknown to himself, deep in his own!

The manner in which food is adulterated is not only one of degree but of kind. The most simple of all sophistications, and that which is most harmless, is the mixture of inferior qualities of the same substance. Indeed, if the price charged were according to quality, it would be no fraud at all, but this adjustment rarely takes place. Secondly, the mixture of cheaper articles of another kind; thirdly, the surreptitious introduction of materials which, taken in large quantities, are prejudicial to health; and fourthly, the admixture of the most deadly poisons in order to improve the appearance of the article "doctored."

The microscope alone is capable of detecting at one operation the nature and extent of the more harmless but general of these frauds. When once the investigator, by the aid of that instrument, has become familiar with the configurations of different kinds of the same chemically composed substances, he is armed with far greater detective power than chemical agents could provide him with. It is beyond the limit of the test-tube to show the mind the various forms of animal and vegetable life which exist in impure water; delicate as are its powers it could not indicate the presence of the sugar insect, or distinguish with unerring nicety an admixture of the common Circuma arrowroot with the finer Maranta. Chemistry is quite capable of telling the component parts of any article: what are the definite forms and natures of the various ingredients which enter into a mixture it cannot so easily answer. This the microscope can at once effect, and in its present application consists Dr. Hassall's advantage over all previous investigators in the same field. The precision with which he is enabled to state the result of his labors leaves no appeal; he shows his reader the intimate structures of a coffee-grain and of oak or mahogany sawdust; and then a specimen of the two combined, sold under the title of genuine Mocha. Many manufacturers and retailers, who have been detected falsifying the food of the public, have threatened actions, but they all flinched from the test of this unerring instrument.

The system of adulteration is so wide-spread and embraces so many of the items of the daily meal, that we scarcely know where to begin—what corner of the veil first to lift. Let us hold up the cruet-frame, for example, and analyze its contents. There is mustard, pepper (black and cayenne), vinegar, anchovy and Harvey sauce—so thinks the unsuspecting reader—let us show him what else beside. To begin with mustard. "Best Durham," or "Superfine Durham," no doubt it was purchased for, but we will summarily dismiss this substance by stating that it is impossible to procure it pure at all; out of forty-two samples bought by Dr. Hassall at the best as well as inferior shops, all were more or less adulterated with wheaten flour for bulk, and with turmeric for color. Vinegar also suffers a double adulteration: it is first watered and then pungency is given to it by the addition of sulphuric acid. A small quantity of this acid is allowed by law; and this is frequently trebled by the victuallers. The pepper-caster is another stronghold of fraud—fraud so long and openly practised, that we question if the great mass of the perpetrators even think they are doing wrong. Among the milder forms of sophistication to which this article is subjected are to be found such ingredients as wheaten flour, ground rice, ground mustard-seeds and linseed-meal. The grocer maintains a certain reserve as to the generality of the articles he employs in vitiating his wares, but pepper he seems to think is given up to him by the public to "cook" in any manner he thinks fit. This he almost invariably does by the addition of what is known in the trade as P. D., or pepper-dust, alias the sweepings from the pepper-warehouses. But there is a lower depth still; P. D. is too Genuine a commodity for some markets, and it is accordingly mixed with D. P. D., or dirt of pepper dust.

In the days of Accum it was usual to manufacture pepper-corns out of oiled linseed-cake, clay, and cayenne pepper, formed into a mass, and then granulated; the fraudulent corns were mixed with the real, to the extent of 17 per cent. This form of imposition, like that of wooden nutmegs among our American friends, has, we are happy to say, long been abandoned. The adulterations we have mentioned are simply dirty and fraudulent, but in the cayenne-cruet we find, in addition, a deadly poison. Out of twenty-eight samples submitted to examination, no less than twenty-four were adulterated with white mustard seed, brick dust, salt, ground rice, and deal sawdust, by way of giving bulk; but as all of these tend to lighten the color, it is necessary to heighten it to the required pitch. And what is employed to do this? Hear and tremble, old Indians, and lovers of high seasoned food—with red lead. Out of twenty-eight samples, red lead, and often in poisonous quantities, was present in thirteen! Who knows how many "yellow admirals" at Bath have fallen victims to their cayenne-cruets? Nor can it be said that the small quantity taken at a time could do no permanent mischief, for lead belongs to the class of poisons which are cumulative in their effects.

He who loves cayenne, as a rule is fond of curry-powder, and here also the poisonous oxide is to be found in large quantities. Some years ago a certain duke recommended the laboring population, during a season of famine, to take a pinch of this condiment every morning before going to work, as "warm and comforting to the stomach." If they had followed his advice, thirteen out of twenty-eight persons would have imbibed a slow poison. Those who are in the habit of using curry, generally take it in considerable quantities, and thus the villainous falsification plays a more deadly part than even in cayenne pepper. Imagine a man for years pertinaciously painting his stomach with red lead! We do not know whether medical statistics prove that paralysis prevails much among "Nabobs," but of this we may be sure, that there could be no more fruitful source of it than the two favorite stimulants we have named.

Our succeeding remarks will fall, we fear, like a bomb upon many a tea table, and stagger teetotalism in its stronghold. A drunkard's stomach is sometimes exhibited at total-abstinence lectures, in every stage of congestion and inflammation, painted up to match the fervid eloquence of the lecturer. If tea is our only refuge from the frightful maladies entailed upon us by fermented liquors, we fear the British public is in a perplexing dilemma. Ladies, there is death in the tea pot! Green-tea drinkers, beware! There has always been a vague idea afloat in the public mind about hot copper plates—a suspicion that gunpowder and hyson do not come by their color honestly. The old Duchess of Marlborough used to boast that she came into the world before "nerves were in fashion." We feel half inclined to believe this joke had a great truth in it; for since the introduction of tea, nervous complaints of all kinds have greatly increased; and we need not look far to find one at least of the causes in the tea pot There is no such thing as pure green tea to be met with in England. It is adulterated in China; and we have lately learnt to adulterate it at home almost as well as the cunning Asiatic. The pure green tea made from the most delicate green leaves grown upon manured soil, such as the Chinese use themselves, is it is true, wholly untainted; and we are informed that its beautiful blush bloom, like that upon a grape, is given by the third process of roasting which it undergoes. The enormous demand for a moderately-priced green tea which has arisen both in England and China since the opening of the trade, has led the Hong merchants to imitate this peculiar color; and this they do so successfully as to deceive the ordinary judges of the article. Black tea is openly colored in the neighborhood of Canton in the most wholesale manner.

Mr. Robert Fortune, in his very interesting work, "The Tea Districts of China and India," gives us a good description of the manner in which this coloring process is performed, as witnessed by himself:—

"Having procured a portion of Prussian blue, he threw it into a porcelain bowl, not unlike a chemist's mortar, and crushed it into a very fine powder. At the same time a quantity of gypsum was produced and burned in the charcoal fires which were then roasting the teas. The object of this was to soften it, in order that it might be readily pounded into a very fine powder, in the same manner as the Prussian blue had been. The gypsum, having been taken out of the fire after a certain time had elapsed, readily crumbled down, and was reduced to powder in the mortar. These two substances, having been thus prepared, were then mixed together in the proportion of four parts of gypsum to three parts of Prussian blue, and formed a light blue powder, which was then ready for use.

This coloring matter was applied to the teas during the process of roasting. About five minutes before the tea was removed from the pans—the time being regulated by the burning of a joss stick—the superintendent took a small porcelain spoon, and with it he scattered a portion of the coloring matter over the leaves in each pan. The workmen then turned the leaves round rapidly with both hands, in order that the color might be equally diffused. During this part of the operation the hands of the workmen were quite blue. I could not help thinking if any green tea drinkers had been present during the operation, their taste would have been corrected, and I believe improved.

One day an English gentleman in Shanghæ, being in conversation with some Chinese from the green-tea country, asked them what reason they had for dyeing the tea, and whether it would not be better without undergoing this process. They acknowledged that tea was much better when prepared without having any such ingredients mixed with it, and that they never drank dyed teas themselves, but justly remarked, that, as foreigners seemed to prefer having a mixture of Prussian blue and gypsum with their tea to make it look uniform and pretty, and as these ingredients were cheap enough, the Chinese had no objection to supply them, especially as such teas always fetched a higher price.

I took some trouble to ascertain precisely the quantity of coloring matter used in the process of dyeing green teas, not certainly with the view of assisting others either at home or abroad, in the art of coloring, but simply to show green tea drinkers in England, and more particularly in the United States of America, what quantity of Prussian blue and gypsum they imbibe in the course of one year. To 14½ lbs. were applied 8 mace 2½ candareens of coloring matter, or rather more than an ounce. To every hundred pounds of colored green tea consumed in England and America, the consumer actually drinks more than half a pound of Prussian blue and gypsum. And yet, tell the drinkers of this colored tea that the Chinese eats cats and dogs, and they will hold up their hands in amazement and pity the poor Celestials.

If the Chinese use it in these quantities to tinge the genuine leaf, how much more must the English employ in making up fresh exhausted leaves! That every spoonfull of hyson or gunpowder contains a considerable quantity of this deleterious dye will be seen by any one who places a pinch upon a fine sieve, and pours upon it a gentle stream of water, when the tinging of the liquid will show at once the extent of the adulteration, and the folly of drinking painted tea. Assam tea, though not so inviting in color, is free from adulteration. A word to the wise is enough.

Of fifty samples of green tea analyzed by Dr. Hassall, all were adulterated. There is one particular kind which is almost entirely a manufactured article—gunpowder, both black and green—the former being called scented caper. Both have a large admixture of what is termed "lye tea," or a compound of sand, dirt, tea-dust and broken down portions of other leaves, worked together with gum into small nodules. This detestable compound, which, according to Mr. Warrington,* who has analyzed it, contains forty-five per cent. of earthy matter, is manufactured both in China and in England, for the express purpose of adulterating tea. When mixed with "scented caper" it is "faced" with black lead; when with gunpowder, Prussian blue; turmeric and French chalk give it the required bloom. Mr. Warrington states that, about 750,000 lbs. of this spnrious tea has been imported into Great Britain within eighteen months? Singularly enough, the low-priced teas are the only genuine ones. Every sample of this class which was analyzed by Dr. Hassall, proved to be perfectly pure. Here at least the poor have the advantage of the better classes, who may pay a higher price to be injured in their health by a painted beverage.

The practice of re-drying used-up leaves, is also carried on to some ex-

*In an article upon the teas of commerce, which appeared in the Quarterly Journal of the Chemical Society for July, 1851.

tent in England. Mr. Geo. Philips, of the Inland Revenue Office, states that in 1843 there were no less than eight manufactories for the purpose of re-drying tea leaves in London alone, whilst there were many others in different parts of the country. These manufacturers had agents who bought up the used leaves from hotels, clubs, coffee-houses, etc., for two-pence-half-penny and three-pence per lb. With these leaves, others of various trees were used, and very fine pekoe still flourishes upon the hawthorn-bushes, sloe-trees, etc., around the metropolis.

After being well mixed with a quantity of *copperas*, this poisonous, imitation green tea, "so largely supplied to country grocers," was no doubt used for adulterating other green teas already dosed with Prussian blue, tumeric, etc. These have found their way into many a country home of small means. When the nephew comes on a visit, or the curate calls of an afternoon, the ordinary two spoonfuls of black are "improved" with "just a dash of green," and the poor innocent gentleman wonders afterwards what it can be that keeps him awake all night.

If the better class of black and green teas* are thus vilely adulterated, the reader may fancy he can take refuge in coffee—alas! in too many cases he will only avoid Scylla to fall into Chaybdis. Coffee, as generally sold in the metropolis and in all large towns, is adulterated even more than tea. Aanalyzes made by Dr. Hassall, of upwards of a hundred different samples of coffee, purchased in all parts of the metropolis before the issuing of the order for the labelling of the packages "chicory and coffee," proved that, in a great number of cases, articles sold as "finest Mocha," "choice Jamaica coffee," "superb coffee," etc., contained, in some cases, very little coffee at all; in others, "only a fifth, a third, half, etc., the rest being made up mainly of chicory. Nothing is more indicative of the barefaced frauds perpetrated by grocers upon the public than the manner in which they go out of their way to puff in the grossest style the most abominable trash. The report of the Sanitary Commission gives many examples of these puffs and announcements, which, we are informed, are kept set up at the printers', and may be had in any quantities. We quote one as an example :—

JOHN ———'S COFFEE.

*The richness, flavor, and strength of which are not to be surpassed.*

Coffee has now become an article of consumption among all classes of the community. Hence the importance of supplying an article of such a character as to encourage its consumption in preference to beverages the use of which promotes a vast amount of misery.

John ———'s coffee meets the requirement of the age; and, as a natural result, the celebrity to which it has attained is wholly unparalleled. Its peculiarity consists in its possessing that rich aromatic flavor, combined with great strength and deliciousness, which is to be found alone in the choicest mountain growths. It may, with perfect truth be stated, that no article connected with *domestic economy* has given such general satisfaction, and the demand for it is rapidly increasing.

John ———'s establishment, both for extent and capability, is the first in the empire. Observe!

Every canister of John ———'s coffee bears his signature, without which none is *genuine.*

---

*Assam tea is the only exception to this rule, but very little of it is imported.

At the end of this puff the analyst places these words—"*Adulterated with a considerable quantity of chicory!*"

More erudite grocers treat us to the puff literary, as in the following instance:—

Rich-flavored coffees fresh roasted daily.

USE OF COFFEE IN TURKEY.

Sandys, the translator of Ovid's Metamorphoses, and who traveled in Turkey in 1610, gives the following passage in his Travailes, p. 51, (edit. 1657). Speaking of the Turks, he says:—"Although they be destitute of taverns, yet have they their coffee houses, which sometimes resemble them. There sit they chatting most of the day, and sip of a drink called coffa, of the berry that it is made of, in little china dishes, as hot as they can suffer it, black as soot, which helpeth, as they say, digestion and procureth alacrity."

This pleasant sample of the puff indirect has also appended to it the naked sentence:

"*Adulterated with chicory, of which not less than half the sample consists.*"

We have said it often happens that the adulterations are adulterated. Chicory is an instance of it. The original fraud is found to have ramified in an endless manner; and Sir Charles Wood will, doubtless, be astonished to hear of the hideous crop of falsifications his most unfortunate order has caused to spring out of the ground.

Immediately the process of transforming chicory into coffee became legalized by the Government, that article came into very extensive consumption, and factories were set up especially for its secret manufacture. The reason for this secrecy may be gathered from the list of articles which are made to subserve the purpose: roasted wheat, ground acorns, roasted carrots, scorched beans, roasted parsnips, mangold-wurzel, lupin-seeds, dog's biscuits, burnt sugar, *red earth*, roasted horse-chestnuts, and above and beyond all, *baked horses' and bullocks' livers.*

In close proximity to the tea and coffee pots stand the milk-jug and the sugar-basin. What find we here? A few years ago the town was frightened from its propriety by a little work entitled "Observations on London Milk," published by a medical gentleman of the name of Rugg, which gave some fearful disclosures relative to the manner in which London milk was adulterated. Dr. Hassall's analyzes go to show that, with the exception of the produce of the "iron tailed cow," none of the supposed defilements really exist, and that the milkman is a sadly-maligned individual. Water is added in quantities varying in different samples from 10 to 50 per cent.; and in the more unfashionable parts of the town *all* the cream is abstracted to be forwarded to the West End. If milk *must* be adulterated in large towns, water is undoubtedly the most harmless ingredient; at the same time it will be seen what a fraud is perpetrated upon the public by selling milky water at 4*d.* a quart.

That the London milking-pail goes as often to the pump as the cow we have no doubt. To bring the diluted goods up to a delicate cream color, it is common to swing round a ball of anatto in the can; and other careful observers and writers upon the adulteration of foodhave detected flour, starch, and treacle. All medical men know that children are often violently disordered by their morning and evening potion—an effect which could not come from the mere admixture of water—and we must confess that we ourselves believe the milkman to be a very wicked fellow.

We are afraid, if we look into the sugar-basin, we shall not find much more comfort than in the milk jug. We refer here to the ordinary brown sugars, such as are generally used at the breakfast-table for coffee. It is scarcely possible to procure moist sugar which is not infested with animalculæ of the acari genus, a most disgusting class of creatures. In many samples of sugars they swarm to that extent that the mass moves with them; and in almost every case, by dissolving a spoonful in a wine glass of water, dozens of them can be detected by the naked eye, either floating upon the liquid or adhering to the edge of the glass. Those who are in the habit of "handling" sugars, as it is termed, are liable to a skin affection called the grocer's itch, which is believed to be occasioned by these living inhabitants of our sugar basins. Horrible as it is to think that such creatures are an article in daily use, we cannot charge the grocer directly with their introduction; the evil is, however, increased by the manner in which he mixes, or "handles" as it is termed in the trade, higher-priced sugars with muscovados, bastards, and other inferior kinds, in which the animalculæ abound. In addition to this foreign animal element, grocers sometimes mix flour with their sugar, and if we are to put any credit in popular belief, sand; but of the presence of this gritty ingredient we have never seen any trustworthy evidence. Nevertheless we have said enough to show that the tea-dealer and grocer do their best to supply the proverbial "peck of dirt" which all of us must eat before we die. Would that we were fed with nothing more deleterious or repulsive! Let us see, however, the base admixtures one is liable to swallow in taking—

A CUP OF TEA *or a* CUP OF COFFEE.

*In the Tea.*

If Green—
- Prussian blue.
- Turmeric.
- China clay or French chalk.
- Used tea leaves.
- Copperas.

If Black—
- Gum.
- Black lead.
- Dutch pink.
- Used tea leaves.
- Leaves of the ash, sloe, hawthorn, and of many other kinds.

*In the Milk.*

- On an average 25 per ct. of water.

- Annatto.

*In the Coffee.*

Chicory.

*In the Chicory.*

- Roast wheat.
- " acorn.
- " mangel-wurzel.
- " beans.
- " carrots.
- " parsnips.
- " lupin-seeds.
- " dog-biscuit.
- " horse-chestnuts.

- Oxide of iron.
- Mahogany saw-dust.
- Baked horse's liver.
- " bullock's liver.

" *In the Milk.*

Treacle.
Flour.
Oxide of Iron.
And other unknown ingredients.

*In the Sugar.*

If Brown—
Wheat flour.
Hundreds of the sugar insect.
If White—
Albumen of bullock's blood.

Water 25 per cent.
Annatto.
Flour.
Treacle.
Oxide of Iron.
And other unknown ingredients.

*In the Sugar.*

If Brown—
Wheat Flour.
Hundreds of the sugar insect.
If White—
Albumen of bullock's blood.

As we perceive the teetotallers are petitioning Parliament and agitating the towns for the closing of public houses, we beg to present them, in either hand, with a cup of the above mixtures, with the humble hope that means will be found by them to supply the British public with some drink a little less deleterious to health, a little more pleasant to the palate, and somewhat less disgusting to the feelings.

